# Object exploration is facilitated by the physical and social environment in center‐based child care

**DOI:** 10.1111/cdev.14161

**Published:** 2024-08-27

**Authors:** Ine H. van Liempd, Ora Oudgenoeg‐Paz, Paul P. M. Leseman

**Affiliations:** ^1^ Department of Development & Education of Youth in Diverse Societies Utrecht University Utrecht The Netherlands

## Abstract

Object exploration is considered a driver of motor, cognitive, and social development. However, little is known about how early childhood education and care settings facilitate object exploration. This study examined if children's exploration of objects during free play was facilitated by the use of particular spatial components (floor, tables, and activity centers) and types of play (solitary, social, and parallel). Participants were 61 children (aged 11 to 48 months and 50.8% boys, socioeconomic levels representative of the Dutch population). Intraindividual variability in children's object exploration was predicted by the use of particular spatial components and the social setting, with small‐to‐medium effect sizes. Solitary and parallel play were positively associated with complex object exploration, especially when sitting or standing at child‐height tables. During social play, object exploration was mostly absent.

**Abbreviations**
AIC: Akaike information criterion
ICC: intraclass correlation coefficient

Exploration is a central learning mechanism in early childhood. Children's object exploration is regarded as a key driver of personal, cognitive, and social development (e.g., Adolph & Hoch, 2019; Babik et al., 2022; Zuccarini et al., 2018). While an increasing number of studies have focused on young children's exploration as related to their motor development and the provided opportunities for exploration in controlled lab situations, mostly focusing on single children or parent–child pairs, less studies have addressed exploration in naturalistic situations such as formal child care where also peers are present (Franchak, 2020). Studying exploration in the naturalistic situation of formal center‐based child care is relevant for several reasons. First, worldwide, an increasing number of children are attending child care already from an early age (OECD, 2023). Second, good‐quality child care could, in theory, offer children multiple opportunities to explore substances, materials, objects, and spaces in an enriched setting that differs in this regard from the average home environment. Third, center‐based child care provides the possibility to study exploration in the social setting of the peer group. Insight into the facilitating (but possibly also impeding) effects of the physical environment and social group setting may inform quality improvement and professional development in child care. In the present study, we examined young children's object exploration in relation to both the physical‐spatial characteristics of the indoor spaces of center‐based child care and the presence of other children. We surmised that the complexity of object exploration could differ by physical and social setting and by the combination of these two environmental factors.

Exploration can be seen as an evolving perception–action system of increasing complexity driven by the child's curiosity and increasing skill to act upon the action affordances offered by the physical and social environment (Franchak & Adolph, 2014; Gibson, 1988). Exploration is a core mechanism of the body–brain system to learn about the physical and social world. Through exploration, children gather embodied multimodal knowledge about the physical and mechanical properties of substances, objects, and spaces, while at the same time, learning to select and control their actions to generate the information that is needed for the specific task at hand (Adolph & Robinson, 2015).

Exploration plays a crucial role in learning about complex concepts in several domains (Babik et al., 2022). By actively exploring characteristics of objects children gradually learn to combine and detect new functionalities of objects, gaining, for instance, embodied knowledge about basic mathematical notions (Ginsburg et al., 2006), and about spatial relations and shape‐based categories underlying vocabulary learning (Oudgenoeg‐Paz et al., 2015; Smith, 2013; Zuccarini et al., 2018). Studies show that infants actively explore relations between objects and surfaces, thereby combining physical, mechanical, and spatial properties of both object and surface, which sets the stage for tool use (Adolph & Franchak, 2017; Lockman & Tamis‐LeMonda, 2021).

Exploration is modulated by children's muscular maturation, motor development, and physical growth, referred to as “enabling conditions” (Adolph & Hoch, 2019). The attainment of motor milestones such as sitting, standing, and walking has a profound impact by opening new perception and action possibilities to explore objects and spaces (Kretch & Adolph, 2017). Studies in laboratory and home settings show that changes in postural control, for instance, being able to sit upright, enable children to engage in more complex interactions with objects and with others (Schneider et al., 2023; Thurman & Corbetta, 2019). Exploration is further guided by what the physical‐spatial structures of the environment offer in terms of simple, complex, and nested affordances, which in turn are specified by the physical, spatial, and mechanical properties of objects and other present materials (e.g., Lockman & Kahrs, 2017; Osiurak et al., 2017).

A recent systematic review found that only a few studies investigated the relation between physical‐spatial characteristics and young children's exploration behavior in center‐based child care (Van Liempd et al., 2020). One study suggests that dividing the playroom into recognizable play areas, with low visual boundaries that enable children to keep visual contact with the caregiver, may encourage young children to explore more fully the space of the playroom (Legendre & Fontaine, 1991). A qualitative study with young children (between 6 and 28 months of age) reported an increased occurrence of positive peer interactions and higher cognitive engagement in well‐defined activity centers compared to other areas in the playroom (Musatti & Mayer, 2011). Another study, using the same sample as the present study, examined children's exploration of large spatial objects, including actions such as crawling under a table, climbing a slide, and sliding down. Exploration was positively related to playing in designated areas such as activity centers (van Liempd et al., 2018). However, these studies focused on children's exploration of larger spatial structures, not on the exploration of objects. To the best of our knowledge, possible relations between focused object exploration and the physical‐spatial characteristics of child‐care settings have not been investigated.

While physical‐spatial and social‐cognition are traditionally separate fields of study (Proulx et al., 2016), a recent unified embodied cognition view on social‐cognition holds that social‐cognition emerges from the need of a person to coordinate object‐focused actions in the immediate environment with others, referred to as the peri‐personal space (Barrett et al., 2022; for a comprehensive review on the relationships among action, space and social‐cognition in humans, see Proulx et al., 2016). In this space, the intrusion of others is initially perceived as a disturbance, which is to be avoided. Accumulating positive experiences that others in the peri‐personal space provide nurturance and comfort—as in typical infant–parent interactions in the first months of life—motivates an approach tendency and coordination of actions (e.g., turns and gestures). Coordination is broadly conceived here as including at least some degree of attention sharing, mutual understanding, and behavioral control (e.g., not interfering with each other's activities), perceiving the consequences of others' actions, imitation of the action goals of others, and collaboration with others for a joint (emerging) goal (Tomasello, 2022). Underlying this is the growing capability of the brain's mirror systems to predict the actions of others as well as the consequences of one's own actions for others (Meyer et al., 2022; Van Elk et al., 2008). This prediction ability is constrained by what the children themselves are already able to do, which depends on the stage of motor development and the previous action experiences of the child (Van Elk et al., 2008). Studies show that children younger than 1 year can already coordinate actions if scaffolded by an adult, while 2‐year‐olds show a growing ability to coordinate their actions in a joint task without adult support (Meyer et al., 2015).

Children's play and similar activities in naturalistic settings, including sensorimotor object exploration, are traditionally viewed within a developmental stage perspective, either along a social dimension or a cognitive dimension (for a review, see Lillard, 2015). Parten (1932) distinguished solitary, onlooker, parallel, associative, and cooperative play, with the latter forms being regarded as more mature. Smilansky (1968), following Piaget's stage theory of cognitive development, distinguished four stages of play: functional sensorimotor play, constructive play as a more mature form of sensorimotor play, dramatic play, and games with rules as the most mature form of play. Rubin and colleagues, in a series of studies, questioned the assumption that the putatively immature (solitary and parallel) forms of play are also (necessarily) cognitively immature (e.g., Coplan et al., 2015; Rubin, 1982). Solitary and parallel play can involve elementary sensorimotor object exploration as well as more complex construction activity, while social cooperative play can still involve simple sensorimotor functional play. Indeed, studies have demonstrated that both solitary and parallel play remain common types of play over the years, both contributing to child development (Coplan et al., 2015; Reikeras, 2020; Xu, 2010). Social play develops with age and becomes more complex and diverse as children's action coordination ability and language proficiency increase. Actions with objects can vary accordingly: objects can be combined during joint construction play (complex exploration) or be used as attributes in symbolic or pretend play (Ramani, 2012; Whitebread & O'Sullivan, 2012).

In the theoretical framework of the current study, children's developing coordination ability enables first solitary, then parallel, and then collaborative exploration. With increasing coordination ability, additional forms of learning through exploration in a social setting become available. Solitary play is often referred to as non‐social (Coplan et al., 2021). In the present framework, solitary exploration in a naturalistic group care setting may reflect an early form of coordination: avoiding interference by others by finding a place to explore the action affordances of objects, while respecting the personal space of others. Object exploration in this situation is driven by curiosity and the options for action afforded by the object and wider physical‐spatial environment. Parallel exploration, enabled by increasing coordination ability, may additionally foster emulation learning from the consequences of other children's exploratory actions upon objects and learning by imitation. Finally, coordination of actions in collaborative social play may create a zone of proximal development that enables children to explore combinations of multiple objects to realize an emerging common goal that none of the children individually would have been capable of (e.g., building together a railroad yard; for an example, see Van Schaik et al., 2018).

## Present study

The aim of the present study was to examine how young children's object exploration is associated with the physical‐spatial and social characteristics of the child‐care setting during free, unguided play. The study involved children between 1 and 4 years of age attending daycare centers in The Netherlands. We focused on free, unguided play in a naturalistic setting to be able to observe children's self‐initiated interactions with the physical‐spatial and social environment without professional caregiver intervention. Free, unguided play is a common curriculum component in Dutch child care (Singer et al., 2014; Slot et al., 2015) as well as in other countries (Fuligni et al., 2012; Rentzou et al., 2019). In a previous study on young children's use of spatial components in center‐based child care, using the same sample as in the current study, three spatial components were found to be used most often: the free floor space, activity centers, and tables (Van Liempd et al., 2018). Therefore, in the current study, we only included observations where one of these components was used. The components not included in the present study were each used less than 6% of the time.

Given the lack of studies addressing object exploration during free, unguided play in child‐care settings, the present study was largely exploratory. Yet, based on the theoretical account outlined above and on previous research, we tentatively hypothesized that complex object exploration involving combinations of several objects (e.g., stacking blocks and making puzzles) would occur more frequently in well‐defined spatial settings, in particular at tables and in activity centers that allowed children to take an upright posture with their hands free (sitting, kneeling, and leaning), enabling them to explore and combine objects in front of them, while no or only simple exploration of objects (e.g., carrying or moving an object) would prevail in less well‐defined areas, such as the free floor space. We furthermore hypothesized that complex object–exploration would be facilitated by the presence of other children through increasing levels of action coordination. For the youngest children, until about age 2, action coordination might appear mostly as avoiding interference while exploring objects, corresponding to solitary play, and as emulation learning and imitation, corresponding to parallel play. In older children, from about age 2, object exploration might increasingly also appear as joint action coordination in combining multiple objects to create a complex construction, corresponding to collaborative social play. Lastly, we expected there could be an optimal combination of spatial and social settings to facilitate complex object exploration.

## METHOD

### Participants

Participants were 61 children (50.8% boys) from 10 child‐care centers, all belonging to a large provider of child care in the Netherlands. The centers were located in 10 villages and small towns, spread across the country. Regarding socioeconomic status, all levels of the current Dutch population were represented. Data collection took place during the Spring of 2016. Due to standardization within the corporate organization and due to compliance of all centers with legal requirements regarding group size, staff‐to‐children ratio, and group composition, the 10 centers were highly similar with regard to their spatial design and furnishings. Tables were mostly at child height and all playrooms had a number of activity centers with play materials within children's reach. All centers complied with the legal regulations regarding number of square meters available per child (≥3.5 m^2^).

In each center, one care group was selected based on two criteria: (1) the group had to function as a mixed‐age group for at least 6 months to avoid disturbing effects of recent major changes in group composition; and (2) each group had to consist of both younger (under 24 months) and older (24–48 months) children to assure that we could recruit enough children from different ages. Since active self‐induced locomotion enables children to choose their own location and social context in which they play, only children who could actually move around without help by crawling or walking were included in the study. In each group, five to seven target children were selected for observation through video recording, such that an even distribution of age and gender was obtained. The mean age of the observed children was 29 months (SD = 9.95; age range: 11 to 48 months). Use of child care varied between 1 and 5 days a week (*M* = 2.20; SD = 1.00). At the time of the study, children had been attending the center on average for 21 months (SD = 10.84) with a range of 1 to 44 months. The total number of children in the groups during the observations ranged from 8 to 11 (*M* = 9.98; SD = 0.88). Informed consent of the parents was obtained for 88% of the children attending the groups. Children for whom no consent was obtained were not included in the study and were temporarily cared for in another group during the video recordings or were carefully kept out of sight by a caregiver when another child was being recorded. Note that the term “caregiver” in this article refers to a professional caregiver in formal child care. The study was approved by the Ethical Review Board of the Faculty of the University of Amsterdam (protocol number: 2015‐CDE‐4107).

### Procedure

Children were observed during unguided free play in the morning. In each group, data were collected on 2 different days, with 1 or 2 weeks between the first and the second visit, but always on the same day of the week. On both days, video recordings were made during two rounds of 30 min. Recordings started with a period of about 10 min to familiarize the children with the researcher and the camera. Following the standard protocol of the Ethical Review Board, when children are indicated to feel uncomfortable with being observed, observation should be stopped. This did not occur. A first, randomly chosen child was video recorded during a continuous episode of 5 min. After 5 min, another child was recorded for a 5 min episode, and so on. After all children were recorded, the second cycle started. In this way, each child was observed during four episodes of 5 min on the 2 days, 20 min in all. A few children (*N* = 7) were absent on the second day. In these cases, an additional child for whom parental consent was obtained was selected to ensure sufficient data per center. After removing interruptions (e.g., because of diapering) and episodes that were not suited for the purpose of the study (e.g., when a child became involved in a caregiver‐led activity), a total of 216 episodes remained for analysis (*M* = 17.5 min per child), with 7% of the episodes being excluded from the analysis.

Coding of the video recordings was done by dividing each 5‐min episode into 10‐s intervals, 30 per episode. After removing 61 intervals that were not suited for the analysis (e.g., when the observed child left the room), in total *N* = 6419 intervals remained. For each 10‐s interval, three types of behavior were coded: level of object exploration, use of spatial components, and play type. If, during an interval, a child switched between play types or locations, the code for the behavior observed during the largest part of the interval was entered. Caregivers were asked not to make any major changes in the room between the two visits. During the first visit, photographs were taken of the room that were compared with the situation during the second visit; no major changes had occurred. Finally, the caregivers were asked to fill out a structured questionnaire about the characteristics of the children participating in the study.

### Measures

#### Object exploration

A newly developed observation instrument was used to code children's exploration of objects and use of spatial components (Van Liempd et al., 2018). Exploration of objects was coded for each 10‐s interval (see Table 1). Object exploration was coded as *simple* when the child was visually examining, carrying or mouthing, picking up, or throwing an object, and as *complex* when multiple objects were combined, such as making a puzzle or stacking blocks, when advanced motor abilities were needed or object‐related action coordination was required (e.g., giving an object to a peer who could use this object). When a child was not using an object, *no object exploration* was coded. Dummy variables were constructed to represent whether or not a particular level of object exploration was observed during an interval.

**TABLE 1:** Exploration of objects.

No object exploration	Simple exploration	Complex exploration
Not in contact with any objects	Mouthing	Stacking (blocks and cups)
	Carrying	Compiling (puzzles, Lego, cup, and saucer)
	Visually inspecting	Hiding (in or behind)
	Shaking, ticking, and banging	Combining (two or more objects)
	Throwing and kicking riding (with a car)	Standing or sitting on (e.g., car and stacked blocks)
	Seizing and putting down	Giving to someone
	Moving, pushing	

#### Use of spatial components

Coding of the use of spatial components at the interval level was based on a list of spatial components that frequently occur in child‐care centers. Components could be movable objects (such as a table, chair, or playhouse) or fixed (floor, activity center, or cupboard). For each interval, we coded whether or not a particular component was used during that interval. In the present study, we focused on the three spatial components that were used most often by the children: tables, the free floor space, and activity centers. The free floor space is an area that is not occupied by any other major spatial component, where children can move around freely or play with loose objects. An activity center is a spatial component that is intentionally set up for a specific activity such as construction play, pretend play, or literacy activities. Intervals where components other than these three were coded were excluded from the analyses.

#### Type of play

Type of play was coded at the interval level using the Play Observation Scale (Rubin, 2001). The scale first differentiates behavior as either play or non‐play. Play behavior is further divided into solitary, parallel, and collaborative social play. Play behavior was coded as *solitary play* when a child was playing on their own, apart from other children, at a distance greater than 1 m, and attention was mostly focused on their own activity. If a child was playing within a distance of 1 m beside or in the company of another child, but not *with* this child, this was coded as *parallel play*. Whenever a child was engaged in an activity together with one or more other children, with a common goal or play theme, behavior was rated as *social play*. Non‐play behavior was coded when a child was unoccupied, waiting, transitioning to another activity, or standing by. Non‐play behavior was not addressed in the current study (see below). Dummy variables were created representing whether or not the distinguished play types were observed during an interval.

#### Intercoder reliability

Intercoder reliability of the observation measures was determined at the 10‐s interval level. Two coders independently coded all intervals of 4 randomly chosen centers out of the 10 similar centers, which amounts to about 40% of all observation data. Cohen's kappa for dichotomous codes ranged between *κ* = .51 (parallel play) and *κ* = .75 (activity center) with a mean value of *κ* = .64, which was considered satisfactory (McHugh, 2012).

#### Child characteristics

Child characteristics were used as control variables and measured by asking the center's caregiver caring for the child on a daily basis to fill out a child profile questionnaire (Veen et al., 2013). This questionnaire contained questions about the child's age, date of enrollment, number of days per week the child attended the center, and scales to assess temperamental characteristics. These scales were derived from the Early Childhood Behavior Questionnaire (Putnam et al., 2006) and the BRIEF Infant Toddler Social and Emotional Assessment (Briggs‐Gowan & Carter, 2001). For the current analysis, three scales were used: the degree of self‐regulation measured by 11 items (negatively indicated by aspects such as impulsiveness, anxiety, and detachment, reverse coded for the present purpose), the degree of social competence measured by 7 items (addressing aspects such as helpfulness, cooperation, and sociability), and the degree of positive interaction with the caregiver measured by 14 items (trusting and feeling safe), all rated by the caregiver. Sample items of these scales are “This child seems nervous, tense or anxious” and “When this child is sad, she will seek comfort from me.” Caregivers were asked to rate to what extent the presented statements were true for the child on a 5‐point scale. The internal consistency of the three scales was satisfactory with Cronbach's *α* = .77, *α* = .68, and *α* = .78, respectively.

### Analytic procedure

Data analysis proceeded in two steps. First, descriptive and correlational analyses were conducted of children's personal characteristics, object exploration, use of spatial components, and types of play. Second, three series of multinomial two‐level (interval and child) regression analyses were conducted with object exploration as multinomial dependent variable (Hox et al., 2010). A preliminary analysis was conducted to determine the variance in observed object exploration at the center level, revealing a small intraclass correlation coefficient (ICC) = .022. Therefore, also given the small sample size at this level (*N* = 10), the center level was not distinguished in the multilevel analyses (Hox et al., 2010). In the first series of analyses, associations of children's object exploration with the spatial components were examined. In the next series, associations of object exploration with the types of play were analyzed. In the third series, associations with interaction terms of the spatial components and the types of play were tested.

## RESULTS

### Descriptive data

Table 2 shows the means and standard deviations of children's temperamental characteristics, observed level of exploration, use of distinct spatial components, and play and non‐play behavior. Caregiver‐rated social competence was on average relatively high (maximum score is 5). Children's self‐regulation and interaction with the caregiver also showed a positive tendency. Self‐regulation correlated significantly with social competence (*r* = .52, *p* < .001) and with interaction with the caregiver (*r* = .67, *p* < .001).

**TABLE 2:** Means, standard deviation, and range for temperamental characteristics (child level), mean proportions of object exploration, use of spatial components, and play behavior (interval level).

Variables	*N*	*M*	SD
Child profile^a^			
Self‐regulation	61	4.06	.48
Social behavior	61	3.75	.38
Interaction with caregiver	61	3.85	.40
Object exploration^b^			
No object exploration	6419	0.38	.49
Simple exploration	6419	0.46	.50
Complex exploration	6419	0.16	.37
Use of spatial components^b^			
Floor	6419	0.38	.49
Activity center	6419	0.18	.39
Table	6419	0.13	.34
Other (cupboard, big play objects, chairs, and bars)	6419	0.31	.46
Play behavior^b^			
Solitary play	6419	0.16	.36
Parallel play	6419	0.21	.41
Social play	6419	0.12	.32
Non‐play behaviors	6419	0.51	.50

^a^ Score range = 1–5.

^b^ Based on dichotomous scores per 10‐s interval, with values 0 (not observed) and 1 (observed).

No object exploration was observed during 38% of the 10‐s intervals (mean proportion *M* = .38), simple object exploration during 46%, and complex object exploration during 16%. The three selected spatial components were used during 69% of the intervals, with use of the free floor occurring most frequently. The use of a variety of other spatial components added up to 31%, but note that none of these components separately was used during more than 6% of the intervals. Play behavior occurred during 49% of the intervals, with parallel play occurring most frequently. Remarkably, during 51% of the intervals, children were observed *not* being engaged in playful activities.

As indicated, the current study focused on object exploration in relation to three selected spatial components and three types of play. Therefore, intervals, where other spatial components were used or non‐play behaviors were observed, were excluded. This selection resulted in a total of *N* = 2179 observation intervals for the subsequent analyses. This further selection also changed the proportions of observed behaviors compared to the proportions reported in Table 2. For the dependent variable object exploration, after selection, no object exploration was observed during 23% of the intervals, while simple object exploration occurred during 47% and complex object exploration during 30% of the intervals.

No significant correlations were found among children's age, gender, number of days per week attending child care and total time being enrolled in the center, and children's temperamental characteristics. Temperamental characteristics, number of days, and gender were not related to the levels of object exploration, types of play, and the use of the spatial components, and were therefore not included in further analyses. Age and time since enrollment in the facility intercorrelated strongly (*r* = .69, *p* < .01), as could be expected. For that reason, only age was included in further analyses. Since the distribution of age was bimodal (*D*(61) = .14, *p* < .05), two age groups were created by median split (Mdn = 27), resulting in a group of 30 children between 11 and 26 months and a group of 31 children between 27 and 48 months.

Based on the selected intervals, we examined the relations among object exploration, use of spatial components, and types of play on the one hand and age group on the other hand, using one‐way ANOVA. The results revealed significant differences by age group in exploration (younger children showed more simple object exploration and older children were more often observed not to explore objects; no significant differences were found for complex exploration), use of spatial components (younger children more often used the free floor space and older children more often activity centers; no differences in table use), and play type (younger children were more often engaged in solitary and parallel play and older children in social play). Effect sizes were small for exploration and spatial components (*η* ^2^ = .01, respectively *η* ^2^ = .02) and small to medium for play type (*η* ^2^ = .05).

**FIGURE 1:**
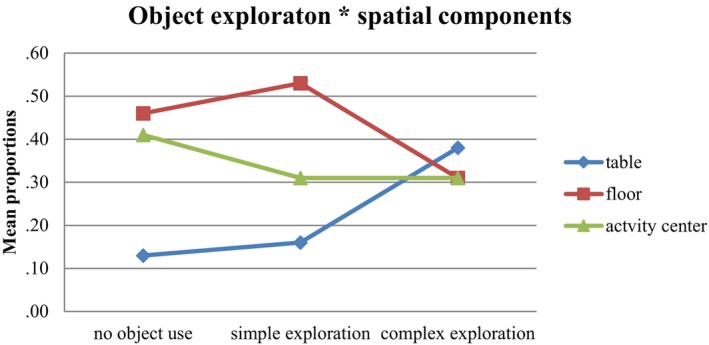
Exploration of objects by using table, floor, or activity center: mean proportions (*N* = 2179).

**FIGURE 2:**
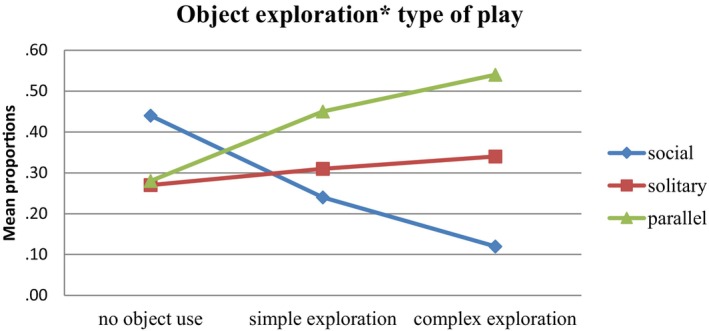
Exploration of objects by type of play: mean proportions (*N* = 2179).

Figures 1 and 2 display the relationship between object exploration and the use of the three spatial components, respectively, the type of play.

Figure 1 shows that, when using the table, complex exploration occurred more often than simple exploration and no object exploration. Simple exploration and no object exploration occurred most often on the floor.

Figure 2 shows that the level of object exploration differed strongly between types of play. During parallel and solitary play, complex exploration occurred more often than during social play.

### Multinomial multilevel analysis of predictors of object exploration

To further examine the relations among object exploration, use of spatial components, and types of play, three series of multinomial multilevel logistic regression analyses were conducted, using MPlus 7.0 (Muthén & Muthén, 2012), distinguishing the interval level (*N* = 2179) and the child level (*N* = 61). In all analyses, the level of complexity of object exploration was the dependent variable. As this variable was coded in three discrete outcomes (no object exploration, simple exploration, and complex exploration), a multinomial logistic regression was conducted, with “no object exploration” as the reference category. Note that in this multinomial regression analysis, the two other categories, that is, simple object exploration and complex object exploration, were compared separately to the reference category (see also Hedeker, 2003).

The three series of multilevel analyses followed the same modeling steps (Hox et al., 2010). First, an intercept‐only model with no predictor variables was defined (Model 1). In this model, the amount of variance in the multinomial outcome measure at the interval level and the child level was estimated, resulting in two ICCs for simple and complex exploration, respectively. Next, a dummy variable for age group (level 2) was added as a predictor, with 0 representing the younger group and 1 for the older group (Model 2). As third step, relevant level 1 predictors were included (Model 3). In the fourth model, the random slopes model, we examined if the relation between the level 1 predictors and the outcome variables varied between children. If the random slope variance at the child level was significant, the model was expanded with cross‐level interaction terms with age group to explore if age group could explain the variance in the relation between object exploration and the level 1 predictors. Finally, to decide which model fit the data best, models were stepwise compared using the deviance difference Δdf index, calculated by comparing the deviance defined by −2 × log‐likelihood, and the difference of the Akaike information criterion with the previous model (ΔAIC). In a multinomial regression analysis, odds ratios, as measures of effect size, are difficult to interpret and thus were not estimated. Instead, pseudo‐*R* ^2^ for the explained variance is provided. Note that the intercept model and the model with age as predictor are exactly the same in all three series of analyses and, therefore, presented only once for the first series (see Table 3).

**TABLE 3:** Multilevel analysis with age and spatial components as predictor of object exploration (*N* = 2179).

Effect	Model 1: random intercept	Model 2: fixed effects: level 1 predictor	Model 3: fixed effects: level 1 + 2 predictors	Model 4: random slopes	Model 5: random slope + cross‐level interactions
	Estimate (SE)	Estimate (SE)	Estimate (SE)	Estimate (SE)	Estimate (SE)
Simple exploration versus no objects					
Fixed effects					
Intercept	0.848** (0.160)	1.163** (0.312)	1.384** (0.268)	0.995** (0.313)	0.864** (0.286)
Age (1 = older group)		−0.609 (0.383)	−0.552 (0.304)	−0.255 (0.381)	0.047 (0.475)
Floor			−0.147 (0.198)	0.240 (0.257)	0.421 (0.388)
Activity center			−0.541** (0.206)	−0.311 (0.417)	−0.243 (0.569)
Floor × age					−0.408 (0.370)
Activity center × age					−0.240 (0.841)
Random effects					
Var simple exploration	1.221** (0.303)	1.090** (0.258)	1.084** (0.279)	1.100** (0.393)	0.954** (0.376)
Var floor				1.552* (0.758)	1.690* (0.799)
Var activity center				2.412* (1.166)	2.032 (1.150)
Complex exploration versus no objects					
Fixed effects					
Intercept	−0.058 (0.223)	0.130 (0.375)	1.321** (0.348)	1.576** (0.260)	1.294** (0.298)
Age (1 = older group)		−0.414 (0.527)	−0.417 (0.437)	−0.903 (0.480)	−0.480 (0.487)
Floor			−1.763** (0.219)	−1.794** (0.394)	−1.387* (0.616)
Activity center			−1.288** (0.220)	−1.526** (0.501)	−1.730* (0.839)
Floor × age					−0.865 (0.855)
Activity center × age					0.356 (1.296)
Random effects					
Var complex exploration	2.457** (0.614)	2.404** (0.612)	2.374** (0.598)	2.161* (0.946)	2.130* (0.944)
Var floor				2.569 (1.443)	2.498 (1.403)
Var activity center				6.356* (2.902)	5.614* (2.676)
Model fit					
Likelihood	−2033.876	−2031.781	−1975.557	1876.900	−1874.887
Deviance	4067.752	4063.562	3951.114	3753.800	3749.774
Diff dev		4.190	112.448	197.314	4.026
Akaike information criterion	4075.753	4075.561	3971.114	3781.800	3785.775
Variance partitioning					
Intraclass correlation coefficient (ICC) simple	.270				
ICC complex	.427				
Pseudo‐*R* ^2^		.001	.029	.077	.078

*Note*: Table is the reference.

* *p* < .05;

** *p* < .01.

#### Spatial components as predictors of object exploration

In the first series of analyses, we tested the hypothesis that complex object exploration would more often occur in well‐defined designated areas, that is, at tables and in activity centers, while no or simple exploration of objects would prevail on the free floor space. Table 3 presents the results of the model testing. Model 1 is the intercept‐only model and shows the variance components at the interval and the child level for simple versus no exploration, respectively, complex versus no exploration. The ICCs show that 27% of the variance of simple object exploration and 43% of the variance of complex object exploration, both relative to no object exploration, could be attributed to the child level. Model 2 includes children's age group as predictor at the child level. Age group was not significantly related to the outcome measures (pseudo‐*R* ^2^ = .001; there was no clear improvement of the model fit based on the Δdf and ΔAIC). Model 3 includes two predictors at the interval level: a dummy variable representing the use of the floor versus the table and a dummy variable representing the use of an activity center versus the table (table is the reference category). The results showed a significantly improved model fit (pseudo‐*R* ^2^ = .03; Δdf = 112.4 and ΔAIC = 104.4).

Model 4 is the random slopes model. The results revealed significant variance at the child level in the effects of the spatial components on the outcome measures, as indicated by significant random effects of the use of the floor and activity centers for simple versus no object exploration and of activity centers for complex versus no object exploration. Allowing random slope effects, increased the model fit substantially (pseudo‐*R* ^2^ = .08; Δdf = 197.3 and ΔAIC = 189.3). To explain the random effects, interaction terms of age group and the two spatial components were added as cross‐level predictors in Model 5; however, the model fit did not clearly improve. Since the random slope variance at the child level of the predictors remained significant or trended toward significance, the random slopes model (Model 4) was considered the final model. The proportion of variance explained in the final model corresponds to a small‐ to medium‐sized effect.

Interpreting the predictive effects of the spatial components in the final model requires integration of all results. First, the occurrence of simple versus no object exploration did not differ between the floor and the table but was significantly lower in activity centers compared to the table. Second, the occurrence of complex exploration versus no exploration was significantly lower both on the floor and in activity centers compared to the table. In combination, the results indicate that use of the table was associated with more complex exploration and use of the floor with simple or no object exploration, as was hypothesized. The use of activity centers was mostly associated with simple or no object exploration, contrary to our hypothesis.

#### Types of play as predictors of object exploration

In the second series of analyses, we tested the hypothesis that types of play involving higher levels of action coordination would be associated with more complex object exploration. Table 4 presents the results of the model testing. Note that Model 1 (the intercept‐only model) and Model 2 (with the child‐level effect of age group) were exactly as in the previous series of analyses (see Table 3) and, therefore, not reproduced in this Table. Model 3 in Table 4 includes two predictors at the interval level: a dummy variable representing solitary versus social play and a dummy variable representing parallel play versus social play (social play was the reference category). The results showed a significantly improved model fit (pseudo‐*R* ^2^ = .03; Δdf = 127.5 and ΔAIC = 119.5). The occurrence of simple versus no object exploration was significantly higher in both solitary and parallel play compared to social play. The occurrence of complex exploration versus no exploration was even stronger and significantly higher in both solitary and parallel play compared to social play. In combination, the results indicated that both solitary and parallel play rather than social play were associated with more complex exploration. There were no clear differences between solitary and parallel play. Contrary to our hypothesis, social play was mostly associated with no object exploration.

**TABLE 4:** Multilevel analysis with age group and types of play as predictors of object exploration (*N* = 2179).

Effect	Model 3: fixed effects: level 1 + 2 predictors	Model 4: random slopes	Model 5: random slopes + cross‐level interactions
	Estimate (SE)	Estimate (SE)	Estimate (SE)
Simple exploration versus no objects			
Fixed effects			
Intercept	0.325 (0.249)	0.305 (0.216)	0.534* (0.262)
Age (1 = older group)	−0.353 (0.313)	−0.397 (0.335)	−0.289926
Parallel	1.127** (0.161)	1.244** (0.240)	0.638 (0.338)
Solitary	0.920** (0.179)	1.138** (0.304)	0.810 (0.444)
Parallel × age			1.131** (0.422)
Solitary × age			0.473 (0.592)
Random effects			
Var simple exploration	1.145** (0.292)	1.261** (0.357)	1.237** (0.345)
Var parallel		0.810 (0.467)	0.512 (0.432)
Var solitary		1.631* (0.715)	1.462* (0.632)
Complex exploration versus no objects			
Fixed effects			
Intercept	−1.499** (0.354)	−1.002** (0.283)	−0.536 (0.354)
Age (1 = older group)	−0.030 (0.449)	−0.479 (0.527)	−0.636015
Parallel	1.984** (0.195)	1.937** (0.316)	1.142** (0.392)
Solitary	1.876** (0.215)	1.343** (0.459)	0.713 (0.690)
Parallel × age			1.403* (0.557)
Solitary × age			0.994 (0.851)
Random effects			
Var complex exploration	2.498** (0.634)	2.380** (0.996)	2.238* (1.061)
Var parallel		1.173* (0.597)	0.950 (0.578)
Var solitary		2906 (1609)	3071 (1673)
Model fit			
Likelihood	−1968.052	−1918.009	−1914.996
Deviance	3936.104	3836.018	3829.992
Δdf	127.458	100.086	6.026
Akaike information criterion	3956.105	3864.018	3865.993
Pseudo‐*R* ^2^	.032	.057	.058

*Note*: Social play is the reference.

* *p* < .05;

** *p* < .01.

Model 4 is the random slopes model. The results revealed significant variance at the child level in the effects of the social setting on exploration. Allowing random slope effects increased the model fit substantially (pseudo‐*R* ^2^ = .06; Δdf = 100.1 and ΔAIC = 92.1). In Model 5, to explain the random effects, interaction terms of age group by solitary play, respectively, and parallel play were added as cross‐level predictors. Although the model fit did not improve, the random variances of parallel play for both simple and complex object exploration and for solitary play for complex object exploration were no longer significant. Therefore, Model 5 was considered the final model. The significant interaction effects on both simple versus no object exploration and complex versus no object exploration suggest that parallel play by older children is even strongly related to the complexity of object exploration. No such cross‐level interaction effect was found for solitary play. The proportion of variance explained in the final model corresponds to a small effect.

#### Spatial components by types of play as predictors of object exploration

In the final series of analyses, we examined to what extent combinations of spatial components and types of play were related to object exploration. Nine dummy variables were created representing interaction terms between all play types and spatial components. Examples are the interaction terms parallel play (vs. other types of play) by table (vs. other spatial components) and social play (vs. other types of play) by activity center (vs. other spatial components). The ninth dummy, representing the interaction term social play by table, was the reference category and not included in the model. The large number of predictors resulted in a complex model that could not be directly estimated. The random slopes model had to be created stepwise by fitting one slope at a time. Next, a model was estimated including the slope variables that showed significant random variance at the child level. Monte–Carlo integration, using 5000 samples, was applied to compute the random slope models (Muthèn & Muthèn, 2012). Table 5 shows the results.

**TABLE 5:** Multilevel analysis with age group and interaction effects of spatial components and types of play as predictors of object exploration (*N* = 2179).

Effect	Model 3: fixed effects: level 1 + 2 predictors	Model 4: random slopes
	Estimate (SE)	Estimate (SE)
Simple exploration versus no objects		
Fixed effects		
Intercept	0.217 (0.395)	0.499 (0.381)
Age (1 = older group)	−0.155 (0.315)	−0.052 (0.469)
Parallel × table	1.579** (0.401)	1.387* (0.622)
Solitary × table	0.920* (0.471)	0.353 (0.452)
Parallel × floor	0.386 (0.386)	1.152** (0.361)
Solitary × floor	1.239** (0.376)	1.381* (0.612)
Social × floor	−0.296 (0.371)	−0.592 (0.942)
Parallel × activity center	0.385 (0.367)	−0.146 (0.419)
Solitary × activity center	0.423 (0.391)	−0.009 (0.449)
Social × activity center	0.250 (0.375)	−0.07 (0.245)
Random effects		
Var simple exploration	1.138** (0.296)	0.915 (0.447)
Var parallel × table		2.375 (2.298)
Var parallel × floor		0.441 (0.546)
Var social × floor		6.653 (3.504)
Var solitary × floor		3.229 (1.905)
Var parallel × activity center		1.201 (1.281)
Complex exploration versus no objects		
Fixed effects		
Intercept	−1.410* (0.578)	−0.397 (0.416)
Age (1 = older group)	0.027 (0.464)	−0.242 (0.612)
Parallel × table	3.001** (0.527)	2.156** (0.714)
Solitary × table	3.197** (0.576)	2.355** (0.505)
Parallel × floor	1.544** (0.531)	0.669 (0.501)
Solitary × floor	1.215* (0.525)	0.074 (0.915)
Social × floor	−0.464 (0.532)	−1.505 (0.861)
Parallel × activity center	1.268* (0.512)	0.412 (0.487)
Solitary × activity center	1.818** (0.529)	0.980 (0.530)
Social × activity center	0.011 (0.536)	−0.698 (0.539)
Random effects		
Var complex exploration	2.658** (0.678)	2.557* (1.208)
Var parallel × table		2.738 (3.183)
Var parallel × floor		0.716 (0.822)
Var social × floor		5.439 (5.426)
Var solitary × floor		4.311 (3.282)
Var parallel × activity center		1.100 (0.777)
Model fit		
Likelihood	−1893.093	−1803.895
Deviance	3786.186	3607.790
Δdf	277.376	178.396
Akaike information criterion	3830.186	3671.789
Pseudo‐*R* ^2^	.069	.113

*Note*: Slopes model with Monte Carlo integration. Social × table is the reference.

* *p* < .05;

** *p* < .01.

Model 3 is the fixed‐effects model with eight dummy variables representing the interaction terms of spatial components and types of play at the interval level as predictors of object exploration (social play by table is the reference category). Compared to Model 2 (see Table 3), the model fit improved substantially by adding the interaction terms (pseudo‐*R* ^2^ = .07; Δdf = 277.4 and ΔAIC = 245.4). Several interaction terms were significantly associated with the outcome variables. Model 4 is the stepwise created random slopes model with the interaction terms as predictors. Allowing the slopes to vary at the child level, substantially improved the model fit (pseudo‐*R* ^2^ = .11; Δdf = 178.4 and ΔAIC = 158.4), while the random slope variance was reduced and for simple object exploration no longer significant. Model 4, therefore, was considered as the final model. Integrating the results, we found that, overall, solitary and parallel play combined with all spatial components were associated with more simple versus no object exploration and, in particular, with more complex versus no object exploration (larger effects) relative to the reference category social play at the table. Social play on the floor or in an activity center was not associated with more simple or complex exploration versus no exploration relative to the reference category. The combination of solitary and parallel play with the table as spatial component was strongly associated with the level of complexity of object exploration. The proportion of variance explained in the final model corresponds to a medium‐sized effect.

## DISCUSSION

The present study examined young children's object exploration in a naturalistic free, unguided play setting in formal center‐based child care. The aim of this study was to examine to what extent the complexity of young children's exploration of objects was associated with their use of three distinct spatial components in the playrooms (tables, free floor space, and activity centers) and three levels of action coordination with other children, characterized in line with traditional play theories as solitary, parallel, and social play. Following recent studies (Babik et al., 2022; Lockman & Tamis‐LeMonda, 2021; Schneider et al., 2023; Zuccarini et al., 2018), we regarded object exploration as a fundamental learning mechanism of the brain–body system, which is driven by the child's spontaneous curiosity and drive to learn, and guided by the action affordances offered by the physical‐spatial and social environment. Although set up as a largely exploratory study, we formulated three tentative hypotheses about the associations between children's object exploration and the spatial and social environment. Below, we will first highlight the main descriptive findings and then discuss the results of the model testing in relation to our hypotheses.

A first finding to be highlighted is that, during scheduled free play time, children's behavior could be coded as some form of engaged, self‐initiated play in less than 50% of the 10‐s intervals, which is observed more often (e.g., Singer et al., 2014; Storli & Hansen Sandseter, 2019). In more than half of the intervals, children were observed merely waiting, standing by, or wandering around. Object exploration (simple or complex) was observed in more than half of the intervals (62%). However, after selecting only the intervals where play was observed, the frequency of simple and complex object exploration increased to 77%. Thus, children in the current sample were found to be engaged in self‐initiated object exploration most of the time while playing.

A second finding concerns the relationship between social play and the complexity of children's exploration. Social‐collaborative play is traditionally assumed to be a more mature form of play, found, among others, to occur in construction play with multiple objects. In the theoretical framework of the current study, social play was regarded as providing children with opportunities to engage in complex collaborative exploration with multiple objects around an emerging joint goal. However, the descriptive results revealed that social play was more strongly associated with no exploration than with complex exploration, seemingly contradicting developmental theory.

To test our explorative hypotheses, three series of multinomial multilevel regression analyses were conducted. The first hypothesis that complex object exploration would occur most frequently in well‐defined spatial settings, that is, at tables and in activity centers, while no exploration and simple object exploration would occur mostly in the free floor space, was only partly supported by the data. The outcomes of the first series of analyses showed that, at the table, children were predominantly engaged in complex object exploration. On the floor or in the designated area of an activity center, children were more likely to be engaged in playing without objects or in simple object exploration. The outcome that complex object exploration did not prevail in the activity centers was unexpected. A possible explanation could be that activity centers, such as a construction area or a pretend play kitchen, are more open to intrusions by others compared to the table, do not automatically (without adult guidance) result in coordinated activities with multiple objects, or elicit symbolic role play without exploring objects. In contrast, when children are sitting on a chair or standing at a fixed spot at a table, they can use their bodies and the table to create a personal space to explore objects. At the same time, this position also allows them to watch other children at the same table and learn from them.

The second hypothesis stated that object exploration would be related to the social dimension of play. We hypothesized that more mature forms of play, such as parallel and social play, compared to solitary play, would offer more opportunities for complex exploration through observation, imitation, and collaborative action coordination. The results of the second series of analysis did not confirm the hypothesis. Both simple and complex object exploration were more frequent during solitary and parallel play than during social play, while no object exploration more often occurred during social play. However, the predictive effects of play type differed between children (significant random slope variance), which could be explained by the interaction effect of play type with age. Overall, younger children were more often engaged in simple and complex object exploration than older children. However, during parallel play, older children were more likely than younger children to engage in complex object exploration. A possible explanation is that, as such, older children compared to younger children have more options to engage in complex object exploration due to their enhanced capabilities to coordinate actions with other children. Parallel play of older children does indeed seem to facilitate more complex object exploration relative to younger children, possibly by providing older children with additional clues to explore objects through emulation learning and imitation. Social play, however, apparently does not automatically elicit more complex joint object exploration but may also, or even predominantly, result in pretend play without exploration of objects.

Lastly, we examined the joint associations between the use of spatial components and the social dimension of children's play. We hypothesized that children's object exploration would be more complex during parallel or social play in well‐defined, designated areas (table and activity center). This exploratory hypothesis was only partly confirmed, reflecting the findings of the previous series of analyses but adding a new insight regarding the combination of well‐defined areas, play type, and age. The outcomes of this final analysis showed that complex exploration of objects mostly occurred during solitary and parallel play at the table, regardless of age. Apparently, combining play types with spatial components could largely explain the remaining random variance in the complexity of exploration at the child level. The pattern of findings may reflect that children in daycare settings can choose when, where, with what, and with whom to engage, older children more so than younger children due to their enhanced ability to coordinate actions with other children. Older children could choose for parallel play at the table, facilitating complex object exploration, as we found, but also for social play on the floor or in an activity center to engage in physical play or pretend play without objects, and, indeed, did this more often, explaining why they overall showed less complex object exploration than younger children.

Two main findings stand out. First, the proposed unified embodied cognition framework to study children's object exploration in the spatial and social context of center‐based child care holds promise by providing the theoretical tools for an integrative account of actions on objects and (object‐related) actions that involve coordination with other children (Barrett et al., 2022; Meyer et al., 2022; Proulx et al., 2016; Tomasello, 2022). The current findings suggest that object exploration in center‐based child care is fostered by providing children with opportunities to explore objects solitarily and in parallel in the well‐defined area of the table (and to a lesser extent in activity centers and on the free floor). The apparent importance of solitary and parallel play in both younger and older children in the present study is in line with studies showing that these types of play remain relevant for cognitive development even as children grow older (Coplan et al., 2015; Reikeras, 2020). The current results add to the still scarce evidence that the interior design and furnishings of the playroom in center‐based child care matters, especially in situations of free, unguided play, and that it is recommendable to have tables at child height that children can freely and independently use to facilitate object exploration (Sando, 2019). The results also indicate that the traditional view of social play as developmentally more advanced than solitary and parallel play may be mistaken from the point of view of learning about the object world.

Second, social play, presumably enabled by a further increased coordination ability and, therefore, more often characterizing older children than younger children, does not facilitate complex object exploration, at least not in situations of self‐initiated free, unguided play in an age group until 4 years of age, as in the current study. In all child‐care centers involved in this study, the activity centers included construction and play kitchen areas for advanced object exploration that would reflect a mature stage of sensorimotor play (Lillard, 2015), yet these activity centers were mostly not used in that way. Perhaps even the older children in this study still needed adult guidance to be able to engage in action coordination with two or more children. This would be in line with earlier studies showing that young children often need verbal instruction or scaffolding by adult caregivers to successfully collaborate with peers (Meyer et al., 2015; Warneken et al., 2014). Other studies in group‐based care, focusing specifically on adult guidance of children's collaborative group processes in joint construction play and pretend play, indeed emphasize the need to scaffold joint goal setting, create a sense of team, and guide collaborative interactions among children (Ereky‐Stevens et al., 2018; Singer et al., 2014; Van Schaik et al., 2018). Thus, the pedagogical format of free, unguided play, as a rather common part of early childhood curricula, may be less suited to foster more mature and challenging collaborative object exploration in groups of young children, despite an enriched and well‐structured spatial environment.

### Limitations and strengths

The first limitation concerns the operationalization of object exploration into only three levels. This is a reduction of the rich information present in the actual situation and may have concealed more subtle differences in the complexity of children's exploration behavior. Likewise, reducing the number of spatial components to three, although representing the most relevant components, and coding just whether these components were used or not, meant a loss of information, for instance, on how these components were used and how exactly the social setting at these components was arranged. These reductions were inevitable within the current quantitative approach, and we believe that, despite these obvious disadvantages, our approach had the advantage that it enabled us to disentangle the complex interplay of key characteristics of the spatial and social setting. In addition, age was included as the only characteristic at the child level. Age was found to correlate strongly with the onset and duration of child‐care attendance, and also with caregiver‐rated temperamental characteristics, therefore this choice could be justified. However, it is relevant to pay more attention to individual differences at the child level in future research. A final limitation of this study could be that it focused on unguided free play and therefore cannot speak to children's exploration under guidance. Given the aim of the current study and the fact that in the Netherlands, as in many other countries, free, unguided play is a core component of the curriculum in early child‐care programs, this focus was justified.

The major strength of the present study is the use of fine‐grained observations and the employment of multilevel modeling, which enabled us to unravel the interplay of physical‐spatial and social factors in relation to children's object exploration and also to test and explain random slope variance at the child level. Most studies in this field have used aggregated data at the individual child level, ignoring intraindividual variation and risking underestimation of the standard errors at the observation level (Hox et al., 2010). Another strength of this study is its ecological validity since it was carried out in a non‐manipulated naturalistic setting, acknowledging that research into child development requires the inclusion of the real contexts in which children are cared for and educated, in addition to controlled lab studies.

## CONCLUSIONS

The current study, involving children between the ages of 1 and 4, showed that self‐initiated solitary and parallel play during scheduled free, unguided play time is associated with complex exploration of objects if taking place at child‐height tables, regardless of the age of the children. Activity centers and the open space of the floor seem less conducive for object exploration and may instead elicit different kinds of play activities. Social play, although theoretically offering additional opportunities to engage in complex coordinated object exploration with peers, was negatively related to the complexity of object exploration, possibly because the social situation elicited other types of play activities (e.g., physical play on the floor and pretend play in activity centers) or because action coordination for an emerging joint goal without adult scaffolding is still difficult for children under 4.

The finding that older children displayed overall less complex object exploration compared to younger children (contradicting developmental theory) could be explained by their choices for locations and types of play that did not yet elicit such complex exploration at this age, which is particularly interesting. This may suggest that developmental progress in naturalistic situations is about enabling conditions (reaching motor milestones, increasing language abilities, and action coordination skills) that give children increasingly more options to choose when, how, with what, and with whom to engage, rather than straightforward progress to putatively more mature forms of sensorimotor exploration in construction play and similar activities. Given the current findings, children's choices are likely also influenced by the specific spatial characteristics of child daycare centers and the presence of other children.

Free, unguided play is a common curriculum component of child daycare in many countries and is thought to contribute to children's development and learning through self‐initiated and self‐regulated play activities, including object exploration. If taking place in a well‐designed and well‐furnished playroom that allows children to play solitarily or in parallel, free play time may indeed contribute to children's development by facilitating object exploration. More complex collaborative object exploration, with potentially enhanced developmental effects, however, likely requires adult scaffolding of action coordination among children at the current young age.

## Data Availability

Data and materials necessary to reproduce analyses are not publicly accessible. The analytic code necessary to reproduce the analyses presented in this paper is not publicly accessible. The analytic code will be made available upon request by emailing the first author. The study's design and analysis were not preregistered.
